# From machine learning to transfer learning in laser-induced breakdown spectroscopy analysis of rocks for Mars exploration

**DOI:** 10.1038/s41598-021-00647-2

**Published:** 2021-11-01

**Authors:** Chen Sun, Weijie Xu, Yongqi Tan, Yuqing Zhang, Zengqi Yue, Long Zou, Sahar Shabbir, Mengting Wu, Fengye Chen, Jin Yu

**Affiliations:** School of Physics and Astronomy, Shanghai Jiao Tong University, Shanghai, 200240 People’s Republic of China

**Keywords:** Optical physics, Analytical chemistry, Cheminformatics, Applied optics

## Abstract

With the ChemCam instrument, laser-induced breakdown spectroscopy (LIBS) has successively contributed to Mars exploration by determining the elemental compositions of soils, crusts, and rocks. The American Perseverance rover and the Chinese Zhurong rover respectively landed on Mars on February 18 and May 15, 2021, further increase the number of LIBS instruments on Mars. Such an unprecedented situation requires a reinforced research effort on the methods of LIBS spectral data analysis. Although the matrix effects correspond to a general issue in LIBS, they become accentuated in the case of rock analysis for Mars exploration, because of the large variation of rock compositions leading to the chemical matrix effect, and the difference in surface physical properties between laboratory standards (in pressed powder pellet, glass or ceramic) used to establish calibration models and natural rocks encountered on Mars, leading to the physical matrix effect. The chemical matrix effect has been tackled in the ChemCam project with large sets of laboratory standards offering a good representation of various compositions of Mars rocks. The present work more specifically deals with the physical matrix effect which is still lacking a satisfactory solution. The approach consists in introducing transfer learning in LIBS data treatment. For the specific application of total alkali-silica (TAS) classification of rocks (either with a polished surface or in the raw state), the results show a significant improvement in the ability to predict of pellet-based models when trained together with suitable information from rocks in a procedure of transfer learning. The correct TAS classification rate increases from 25% for polished rocks and 33.3% for raw rocks with a machine learning model, to 83.3% with a transfer learning model for both types of rock samples.

## Introduction

It is generally considered that the matrix effects, both the chemical^1^ and the physical^2^ matrix effects, represent a critical issue in analysis with laser-induced breakdown spectroscopy (LIBS) for either qualitative classification or quantitative determination^3^. Suitable solutions with respect to such consideration become paramount for applications as important as LIBS analysis of rocks in Mars explorations^4^. The scientific goals, searching for present and past water activities and the traces of the life as well as studying the habitability of Mars^5–7^, rely, at least partially, on the reliability and the accuracy of the analytical data that one can extract from the LIBS spectra recorded by LIBS instruments embarked on Mars rovers^8^. The diversity of chemical compositions of Mars rocks has been studied in previous missions, the absence of real sample from Mars, except meteorites, requires a large number of laboratory rock standard samples in order to cover the expected chemical variety of Mars rocks. It was the purpose of the sets of laboratory standard rock samples prepared and used by the ChemCam team for training and validation of the Mars LIBS spectral data processing models. The number of the involved samples was first 69^9^, and was further increased to 408 in order to offer a more complete representation of the chemical and mineral compositions of Mars rocks^10^. It is important to point out that all the above mentioned laboratory rock standards were prepared in the forms of pressed powder pellet, glass, or ceramic to minimize the heterogeneity and the surface roughness of the samples in the scale of LIBS observations of typically several hundred μm. Such sample preparation leads to obvious differences in surface physical properties between laboratory standards and real rocks analyzed by LIBS instruments on Mars. From these differences, changes in the spectra can rise (physical matrix effect) which can impact the analytical results. With this concern, the effects of sample surface asperity on the hydrogen emission line has been investigated^11^. Our recently published work^12^ observed and analyzed the performance of a machine learning-based model^13^, trained with a set of pressed rock powder pellets for total alkali-silica (TAS) classification^14^ of rocks in their natural state. A significant degradation of the model prediction performance compared to the prediction for pellet samples has been observed. Such degradation prevents the models trained with laboratory standards from reliable predictions with LIBS spectra acquired on raw rock samples, a situation that can lead to misinterpretations for in situ LIBS analysis of rocks on Mars, since we are not yet able to bring materials back from Mars.

In order to search a solution for the issue raised, this work introduced transfer learning in LIBS spectral data treatment to more specifically overcome the physical matrix effect. Transfer learning is considered in machine learning when the knowledge gained while solving one problem is required to be applied to a different but related problem^15^. Its necessity comes from the fact that a major assumption in machine learning data processing is that the training and the model-targeted samples to be analyzed should share the same feature space and have the same distribution^16^. It is unfortunately not the case for the application scenario that we consider. Moreover, transfer learning has recently emerged as a new learning framework to address the problem of insufficient training data in an application (target domain) with the help of the knowledge learnt from a related application having the capability to get sufficient training data (source domain)^17^. Such strategy fits well the requirement of LIBS analysis of rocks on Mars, where sufficient laboratory standards can be prepared as the source domain, whereas real Mars rock samples are not yet available as the target domain. Simulation of their chemical as well as physical properties by terrestrial materials, whether natural or artificial, appears therefore a suitable solution. According to the specific contents of the “knowledge” to be transferred, we can distinguish feature-representation-transfer, where parts of relevant features respectively from the both source and target domains are merged and selected for their low sensitivity to the difference between the two domains, to form a common set of features contributing to the training of a transfer learning model^18,19^. Instance-transfer is another specificity of transfer learning where data of the samples from the both source and target domains participate in the model training, with a conditional testing on the relevance of each sample from the source domain for its effectiveness in improving the performance of the model in a cross-validation process with the data from the target domain^18,19^. A weight is then applied to each source domain sample participating the training, according to its contribution in improving the performance of the model for predicting with target domain data. We note that algorithms belonging to transfer learning, low rank alignment of manifolds or feature-based transfer learning for example, have been used respectively for calibration transfers between different LIBS instruments^20^ or metallic samples with different temperatures^21^.

More specifically, in our experiment, on the basis of the LIBS spectra acquired from a set of laboratory standard samples in the form of pressed powder pellet, machine learning-based multivariate models were trained, validated and then used to predict the concentrations of major oxides necessary for TAS classification of rocks, SiO_2_, Na_2_O and K_2_O, with LIBS spectra acquired from natural rocks. The purpose was first to observe the physical matrix effect due to the difference in surface states between pressed powder pellets and rocks. Since for a LIBS measurement, such difference can be in particular due to the surface hardness, heterogeneity or roughness of a rock, the rock was thus measured in its raw state and with a polished surface, in such way that the different contributions to the physical matrix effect can be investigated separately. Transfer learning-based models were trained with the implementation of feature-representation-transfer and instance-transfer to effectively correct the physical matrix effect in the concentration prediction for rocks in their raw state or prepared with a polished surface, allowing their satisfactory TAS classifications. The correct TAS classification rate increases from 25% for polished rocks and 33.3% for raw rocks with a machine learning model, to 83.3% with a transfer learning model for the both types of rock samples.

## Samples, experimental setup and measurement protocol

### Samples

**Figure 1:**
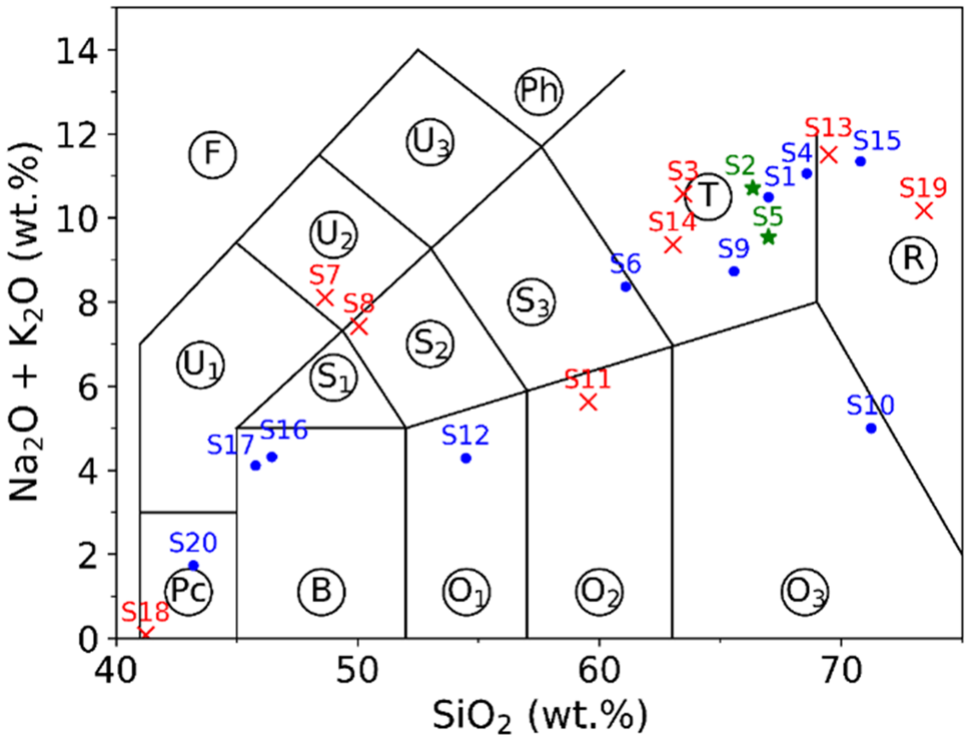
Presentation of the used rock samples in a TAS diagram according to their major oxide concentrations determined using XRF. The short notations of the 15 fields (surrounded by circle) are according to Reference^22^. The green stars represent the 2 isolated samples for model validation; the blue dots represent the 10 additional validation samples for the transfer learning models, and the red crosses represent the 8 training rock samples for the transfer learning models.

In this work, 20 natural terrestrial rocks were used as samples for LIBS analysis. The rocks were first washed using alcohol and distilled water before any further treatment. All the rocks were prepared in 3 different forms. Raw rocks: LIBS measurements took place on the natural surface of each rock; Polished rocks: LIBS measurements took place on a polished flat surface of each rock (prepared using a 300-mesh sandpaper); Pellets: a part of each rock was crushed and ground into a powder by a laboratory mill and then sieved by a 300-mesh screen (grain size < 50 μm). A binder (microcrystalline cellulose powder) with a similar particle size was mixed into the rock powder at a weight ratio of 20%. One gram of the obtained mixture powder was pressed under a pressure of 850 MPa for 30 min to form a pellet of 15 mm diameter and 2 mm thickness. The composition, with especially the concentrations of major oxides, SiO_2_, Na_2_O, K_2_O, of each rock was determined with X-ray fluorescence spectroscopy (XRF) performed on the pellets with large enough analyzed area to get their bulk composition. The detailed compositions and the geological names of the rocks are presented in the section “Methods” (Table 4), which allows presenting the rocks in a TAS diagram as shown in Fig. 1. The short notations of the 15 fields (surrounded by circles) are according to Reference^22^ In order to better simulate an application where the samples to be characterized are not available as in the case of Mars exploration, we first isolated 2 samples (S2 and S5) for model validation, they were excluded from the model training processes. Although these 2 samples were randomly selected as typical isolated validation samples, later, for the model performance evaluation, we will involve other pairs of validation samples excluded from the model training process in order to obtain average model performances independent on the choice of validation samples. The corresponding pellets of these 2 samples were used to validate the machine learning model trained using the rest 18 pellets, while the rock forms of these 2 samples joined the rock validation samples in the validation of the transfer learning models, without counterpart pellet in the training sample set. Among the 18 remaining samples, 8 rocks were selected as training samples (S3, S7, S8, S11, S13, S14, S18 and S19). They joined the 18 pellet samples in the training process of the transfer learning models. The rest 10 rocks, together with the above 2 isolated rocks, ensured the validation of the transfer models. In Fig. 1, the 2 isolated samples are shown in green stars, the 10 additional validation samples in blue dots, and the 8 training rocks samples in red crosses.

### Experimental setup

**Figure 2:**
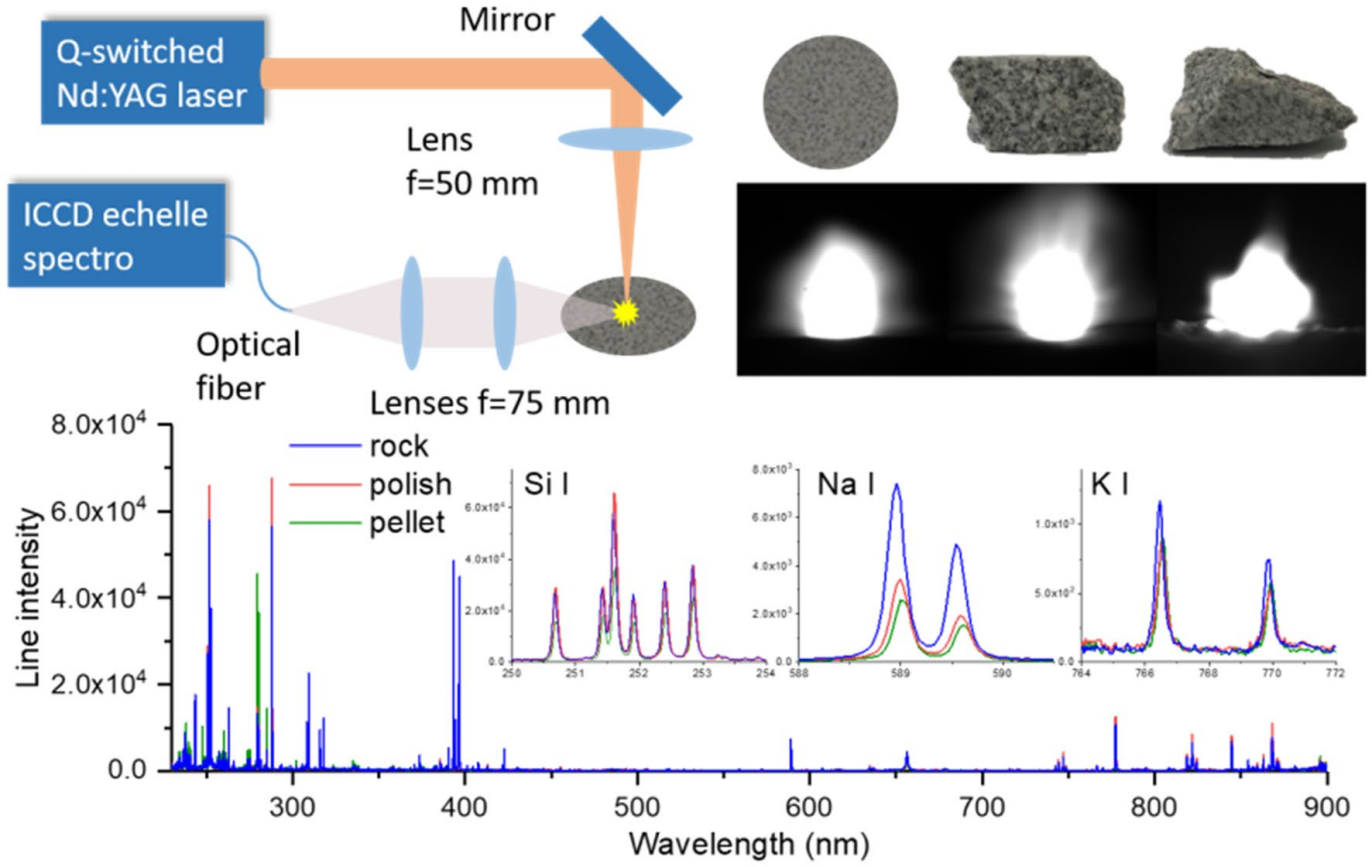
Schematic presentation of the used experimental setup, together with integrated plasma images respectively induced on a pellet, a polished rock and a raw rock of a typical sample (S1), and the corresponding replicate-averaged raw spectra showing differences in average emission intensities of Si, Na and K between a pellet and the corresponding polished and raw rocks.

A detailed description of the used experimental setup can be found elsewhere^12^. Briefly as shown in Fig. 2, a Q-switched Nd:YAG laser operated at a wavelength of 1064 nm with a pulse duration of 7 ns and a repetition of rate of 10 Hz, was used to ablate the samples with a pulse energy of 8 mJ. A lens of 50 mm focal length focused laser pulses about 0.86 mm below the surface of a sample. The diameter of the laser spot on the sample surface was estimated to 150 μm, leading to a laser fluence on the sample surface of about 45 J/cm^2^, or an irradiance of about 6.5 GW/cm^2^. The emission from a generated plasma was collected by a combination of two quartz lenses with a same focal length of 75 mm into an optical fiber of 50 μm core diameter. The output of the fiber was connected to the entrance of an echelle spectrometer equipped with an ICCD camera (Mechelle 5000 and iStar, Andor Technology) which provided a wide spectral range from 230 to 900 nm with spectral resolution power of 5000. The ICCD camera was triggered by laser pulses and set with a delay and a gate width of respectively 500 ns and 2000 ns. A lateral CCD camera (not shown in the figure) allowed capturing time-integrated plasma images as shown in the inset of Fig. 2. Samples were mounted on a 3D translation stage allowing recording replicate spectra on a sample surface with an ablation crater matrix, while keeping a constant distance between the focusing lens and the sample surface (approximately for a raw rock).

We can see in Table 1 that the mean intensities exhibit different values for different sample forms even though the same sample were concerned, which correspond to biases introduced by the physical matrix effect. In addition, for a given emission line, the *RSD* value generally increases from the pellet to the corresponding polished and raw rocks. This observation indicates that starting from an initial spectral intensity fluctuation of a pellet sample, the fluctuation increases for the corresponding polished and raw rocks due to heterogeneity and then surface asperity. The most important information from the table is that the *RSDs* calculated over all the 3 sample forms are significantly larger than the values calculated for a given sample form. This means that the physical matrix effect due to change of sample form represents the dominant variability of spectral intensity, much more important than usual fluctuations observed when measuring a heterogeneous material such as a rock, confirming the above observation of the biases on the mean intensities.

**Table 1:** Mean value, standard derivation (*SD*) and relative standard deviation (*RSD*) of the intensities of the Si I 251.6 nm, Na I 589.0 nm, and K I 766.5 nm lines, recorded from sample S1 in the 3 the forms of pressed pellet, polished and raw rocks, as well as calculated over all the replicate spectra of the 3 sample forms.

Emission lines	Mean value, standard deviation and relative standard deviation											
	Pellets			Rocks						Over all the 3 sample forms		
				Polished			Raw					
	*Mean*	*SD*	*RSD*	*Mean*	*SD*	*RSD*	*Mean*	*SD*	*RSD*	*Mean*	*SD*	*RSD*
Si I 251.6 nm	38,064	4187	11%	68,410	6841	10%	61,762	8029	13%	56,040	14,010	25%
Na I 589.0 nm	2511	1130	45%	3514	2249	64%	7463	2836	38%	4508	3426	76%
K I 766.5 nm	845	566	67%	849	841	99%	1205	1048	87%	969	1550	160%

## Data treatment method

**Figure 3:**
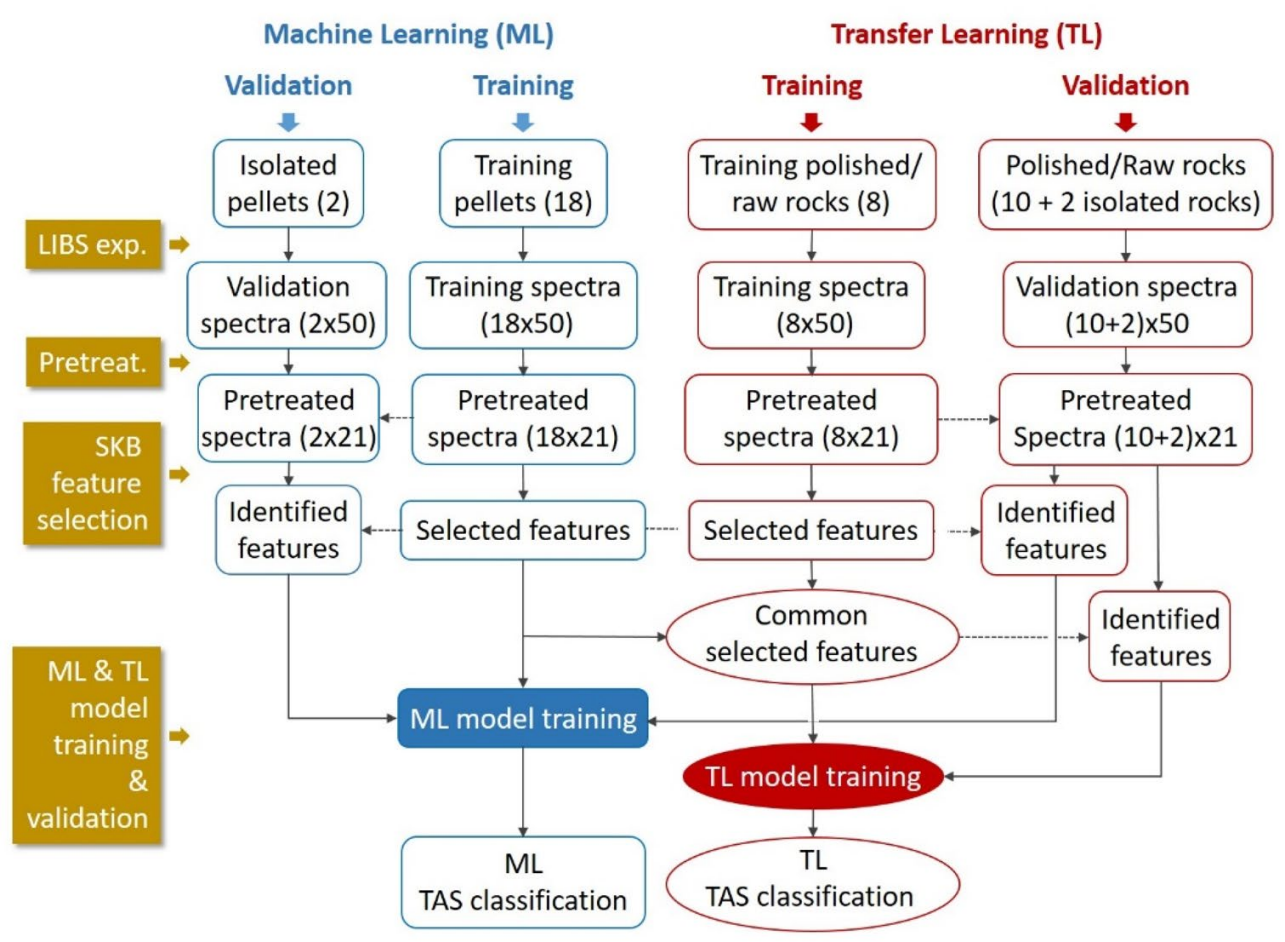
General flowchart used in this work allowing a comparative study between the performances of a machine learning (ML) model and those of a transfer learning (TL) model.

The general data treatment flowchart used in this work is shown in Fig. 3. It was respectively applied to pairs of sample types, pellets/polished rocks and pellets/raw rocks. Several steps can be distinguished: data pretreatment, feature selection, machine learning (ML) and transfer learning (TL) model trainings, as well as model validation. Such flowchart allowed a comparative study between the performances of a machine learning (ML) model and those of a transfer learning (TL) model. As mentioned above, for the machine learning model, the 18 training pellets were used as the training samples set. The resulted model was validated by the 2 isolated pellets as well as the 12 validation rocks including the 2 isolated rocks without counterpart pellet in the training sample set. For the transfer learning models, the training sample set was composed by the 18 training pellets and the 8 training rocks. The resulted models were validated by the 12 validation rocks including the 2 isolated rocks without counterpart pellet in the training sample set.

### Data pretreatment

The pretreatment consisted in the following operations. (i) Average in order to reduce experimental fluctuations and the influence of sample heterogeneity: For each sample, the 50 raw replicate spectra on each sample were averaged in a procedure where an averaged spectrum was calculated with a first group of randomly selected 30 spectra. The remaining 20 spectra then replaced one by one, a spectrum in the first group, each time the new group of 30 spectra was averaged to generate 20 other average spectra. 21 average spectra were generated for each sample. (ii) Baseline correction: an average spectrum was decomposed into a set of cubic splines of undecimated wavelet scales, the local minima were found, then the spline function was interpolated through the different minima to construct the spectral baseline which was removed^23^. (iii) Normalization: baseline-corrected average spectra were normalized with their respective total intensity (the area under the spectrum). (iv) Standardization: standard normal variate (SNV) transformation was respectively applied to the normalized and baseline-corrected average spectra of the training set of the pellet samples (18 × 21 = 378 spectra) and the training set of the rock samples (8 × 21 = 168 spectra). Within a given sample set, for each channel in a spectrum (22,161 channels in total), the variation range of the intensity value over all the samples was transformed into a range with a mean value equal to 0 and a standard derivation (*SD*) equal to 1. The parameters determined in the standardization of the training sets of the pellet and rock samples (the means and the *SD*s) were respectively applied to the 2 isolated pellet samples (2 × 21 = 42 spectra) and the validation rock samples (12 × 21 = 252 spectra) by assuming a same statistical distribution of the data for all the pellets or rock samples. The ensemble of the above operations generated pretreated spectra.

### Spectral feature selection

SelectKBest (SKB) algorithm^24–26^ was respectively applied to the pretreated spectra of the training pellet and training rock sample sets, and successively for the 3 concerned oxides. Within a sample set, for each spectral channel, covariance was calculated between the channel intensity and the concentration of the concerned compound in the corresponding sample, over all the spectra of the sample set. A score was then calculated as a function of the covariance according to the definition given in Reference^13^. A ranking index, $\rho_{i,j}$, was thus associated to each spectral channel according to its obtained score, with 2 indexes $(i,j)$ and a value varying from 1 to 22,161, which ranks the channels from the lowest score to the highest one. Such procedure was applied to the 2 sample sets ($i = 1$: training pellets, $i = 2$: training rocks) and the 3 concerned oxides ($j = 1$: SiO_2_, $j = 2$: Na_2_O, $j = 3$: K_2_O). A feature selection procedure identified 100 highest ranked spectral channels respectively for each of the 3 oxides in each of the 2 training sample sets. Pearson’s correlation coefficient^27^ related to the above mentioned covariance was calculated for the 6 groups of 100 selected features. The results showed that all the selected features had a Pearson’s coefficient larger than 0.75.

As we can see in the Fig. 3, the 3 groups of 100 features selected for the 3 oxides for the training pellet sample set were directly used to respectively train the calibration models for the 3 oxides base on a back-propagation neural network (BPNN). The training algorithm that involved stochastic gradient descent (SGD) and mini-batch stochastic gradient descent (MSGD) optimization iterations, as well as cross-validations with randomly generated statistically equivalent data configurations, has been presented in detail in Reference^13^.

**Figure 4:**
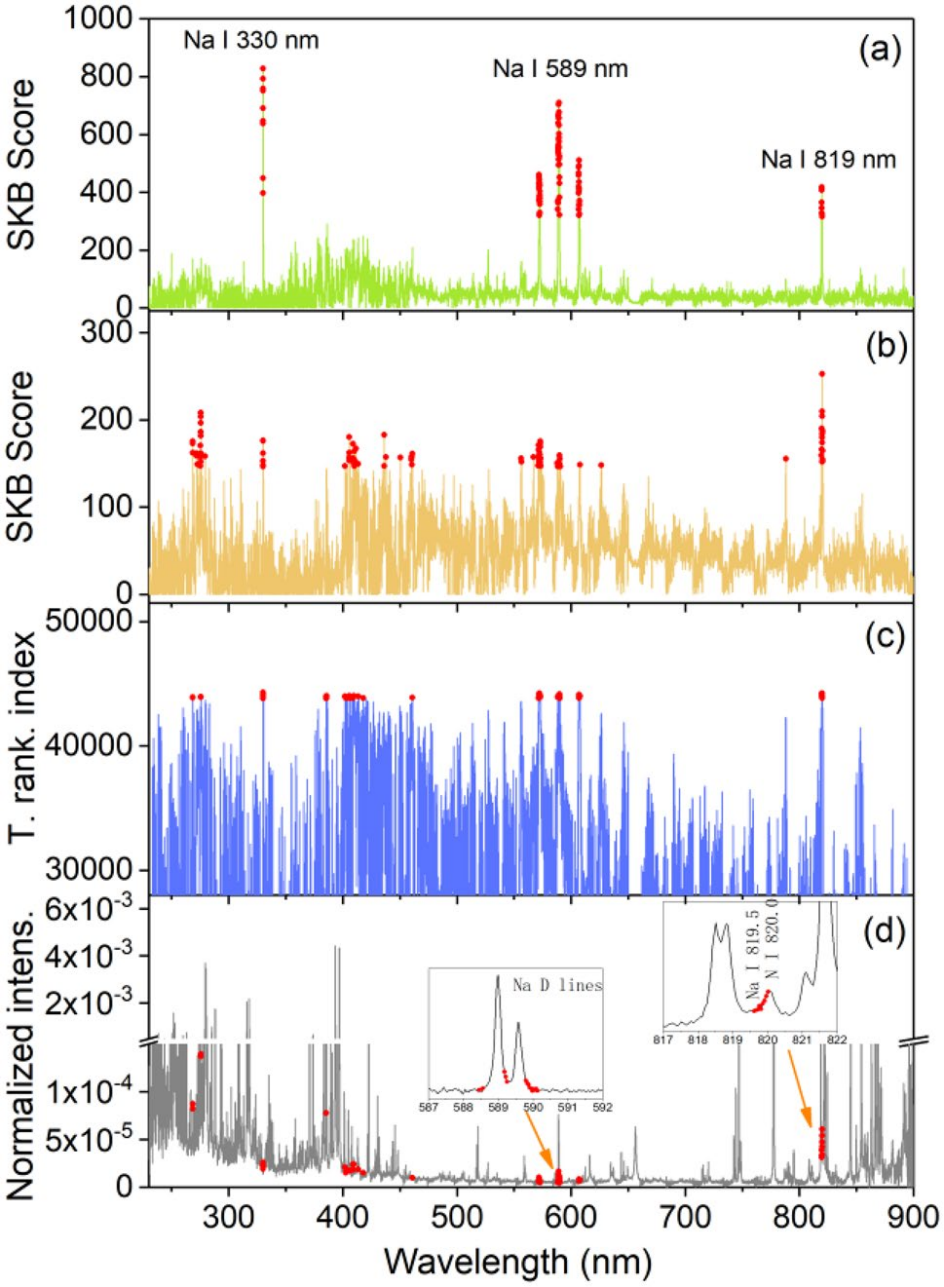
Results of feature selection for Na_2_O: (**a**) for pellets and (**b**) for training raw rocks, SKB scores of all the spectral channels, and the 100 selected features in red dots; (**c**) total ranking index of all the spectral channels and those of the 100 common selected features in red dots; (**d**) a typical normalized average spectrum from sample S1 in pellet form, together with the 100 common selected features in red dots, with 2 insets showing enlarged parts of the spectrum around 589 nm and 820 nm.

For transfer learning model training, and according to the principle of feature-representation-transfer discussed above, an ensemble of common selected features was identified between the training pellet and the training rock sample sets, by calculating a total ranking index $\rho_{j} = \rho_{1,j} + \rho_{2,j}$. One hundred highest ranked features according to the value of $\rho_{j}$ from the highest one to the lowest one, were retained as the common selected features, respectively for the 3 oxides. These groups of features were then fed into the transfer learning model training algorithm. The results of feature selection for Na_2_O for the pair of sample types pellet/raw rock, are shown in Fig. 4. Similar behaviors can be observed in the feature selections for the other 2 oxides and with the 2 pairs for the sample types pellet/raw rock and pellet/polished rock, the corresponding results are shown in the section “Methods” in Figs. 10 and 11.

In Figs. 4a, we can see that for the training pellets, the spectral channels with high SKB scores are clearly concentrated around the several Na emission lines: Na I 330.24 nm and 330.30 nm lines, Na I 588.99 nm and 589.59 nm lines (with 2 groups of ghost lines around 572.1 nm and 606.9 nm), Na I 818.33 nm and 819.48 nm lines. For the training raw rocks in Fig. 4b, the selected features are distributed also among other channels with a significant decrease of the scores for all the important features. This means that the physical matrix effect perturbs the inherent correlation between the emission line intensities of an element and its concentration in the material, and reduces therefore the importance of the line intensities in the concentration determination. At the same time, other spectral channels, such as those around 275 nm and between 410 and 460 nm, get relatively higher scores. This means that they become important in the determination of elemental concentration when using a model based on the training set of the rock samples. These features, representative of the rock samples, are thus included in the common selected features for transfer learning model training. Figure 4c shows in red dots, the total ranking index of the 100 common selected features for Na_2_O. These features are indicated in a typical spectrum in Fig. 4d in red dots. We can see that, beside the features related to the Na emission lines, some features important for the rock samples are included. A more detailed peak identification using the NIST database^28^, shows the contributions from Fe II 268.475 nm and 275.57 nm lines, Si II 385.366 nm and 385.602 nm lines, and the probable contributions from K I 404.414 nm and 404.721 nm lines, Ca I 409.85 nm lines, and Si II 412.807 nm and 413.089 nm lines. A selected feature around 461 nm cannot have easy interpretation.

In the insets of Fig. 4d, 2 parts of the spectrum are enlarged. The inset around 589 nm shows the sodium D lines together with the selected features in red dots. We can see that the selected features are located in the side parts of the line profiles, whereas the central parts of the lines are not retained by the feature selection algorithm. This might be due to self-absorption of the strong resonant Na D lines, which affects much more the central part of the spectral lines. It would also be the reason for the higher scores observed for the weaker Na emission lines around 330 nm. This observation would show the ability of the feature selection process to reduce the influence of self-absorption by selecting the most suitable features inside of a line profile. The second inset in Fig. 4d shows an enlarged part of the spectrum around 820 nm, where we can see the selected features related to the Na I 819.5 nm line in red dots. Due to the spectral interference with the N I 820.0 nm line, only the short wavelength part of the spectral profile around 820 nm is included in the selected features, showing the effectiveness of the feature selection to avoid the influence of spectral interference.

### Transfer learning-based calibration model training

**Figure 5:**
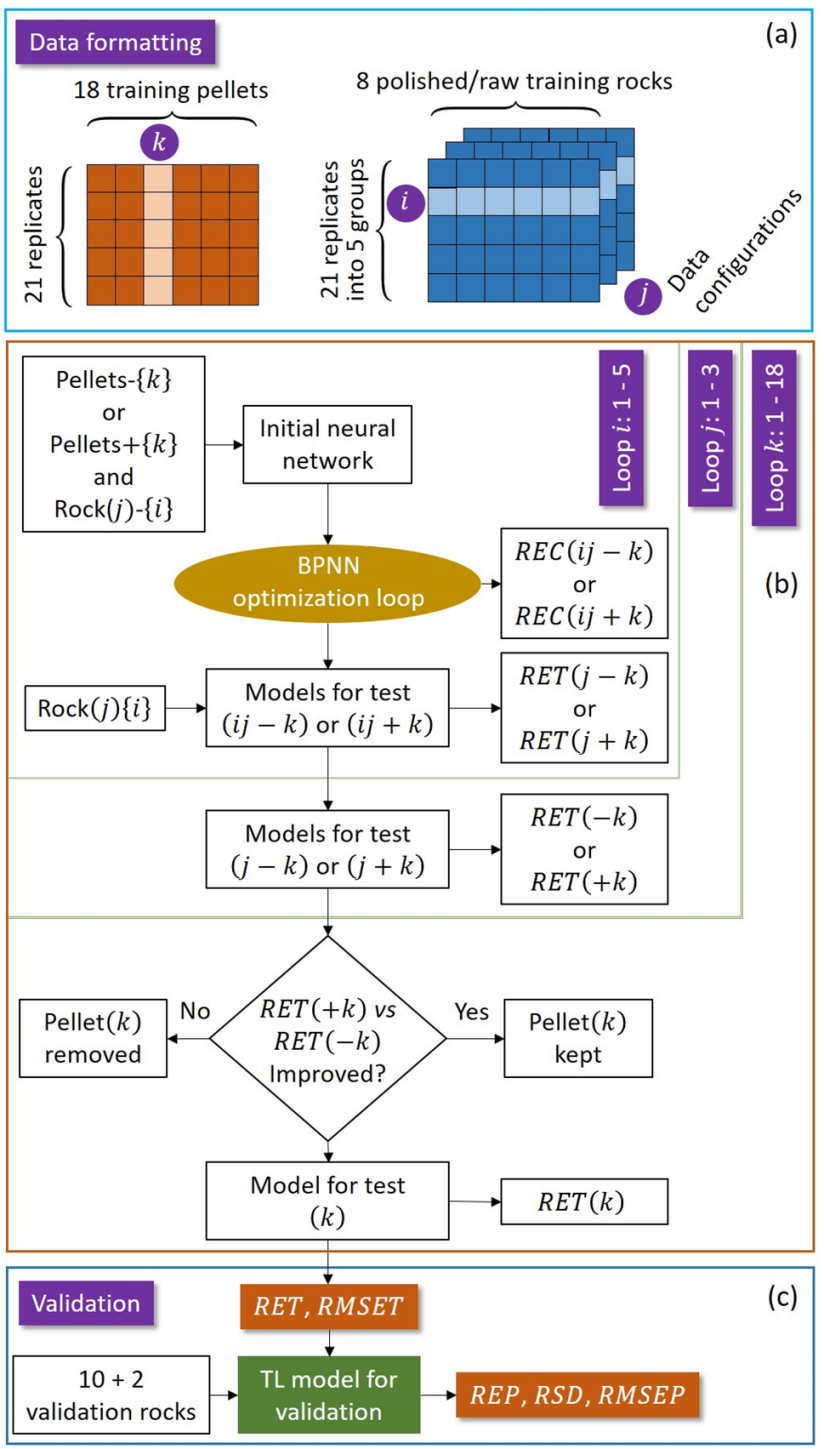
Flowchart of transfer learning model training with the implementation of feature-representation-transfer and instance transfer.

A transfer learning model training algorithm was developed in this work on the basis of that used for machine learning model training presented in detail in our previous publication^13^ and used for various application scenarios^12,29–33^. The flowchart of transfer learning model training is shown in Fig. 5. We can distinguish 3 main steps: data formatting, model training by optimization through iteration loops, and model validation. Training was performed for the 2 pairs of sample types, pellets/polished rocks and pellets/raw rocks and the 3 concerned oxides.

Optimization and assessment of the models were performed in this work using a certain number of indicators specified below: determination coefficient of a regression $r^{2}$ indicating the correlation of the training data with the regression model, limit of detection *LOD* of a model, average relative error of calibration *REC* (%) assessing the trueness of a calibration model to be tested, average relative error of test *RET* (%) assessing the trueness of a model to be tested, average relative error of prediction *REP* (%) assessing the trueness of the model-predicted concentrations for the validation samples, average relative standard deviation *RSD* (%) assessing the precision of the model-predicted concentrations for the validation samples. The mathematical definitions of these parameters can be found elsewhere, in particular in References^13,34^. Moreover, root-mean-square error (*RMSE*) is also used to assess the trueness of the model-predicted values for the training data (*RMSEC*), cross-validation test data (*RMSET*) and validation data (*RMSEP*).

#### Data formatting

According to the above discussed principles of feature-representation-transfer and instance transfer in transfer learning, spectra from the 18 training pellet samples (the source domain) and those from the 8 training rock samples (the target domain), with their 100 common selected features, participated in the training process. These spectra were organized in a given data configuration where the replicate spectra for each sample were arranged in an arbitrarily given order. The effectiveness of each training pellet was tested within an iteration loop where the *RET*s with and without the spectra from the pellet were compared in order to decide the exclusion or the definitive inclusion of the pellet in the final transfer learning model training sample set. It was why the ensemble of pretreated replicate spectra associated to one of the 18 training pellets was indexed with *k* that went from 1 to 18 (Fig. 5a). The 8 training rock samples contributed to the transfer learning model training and in particular, were used in a cross-validation process during the optimization of the neural network. It was why the corresponding spectra were first organized in different data configurations where each configuration *j* corresponded to a certain arrangement of pretreated replicate spectra for each training rock (Fig. 5a). The data configurations were all statistically equivalent since the order of a replicate spectrum of a sample was a dummy index. The number of different data configurations were limited to 3 in this work because more configurations did not bring further improvement of the model as tested in the experiment. For a given configuration *j*, the pretreated replicate spectra of each sample were further organized into 5 groups containing respectively 4, 4, 4, 4 and 5 spectra, respectively. A new index *i* was introduced to designate ensemble of the groups of pretreated replicate spectra of all the training rocks as shown in Fig. 5a. In the model training process, the index *i* went from 1 to 5 within an iteration loop of cross-validation, indicating each time the validation ensemble of the groups of pretreated replicate spectra.

#### Model training by optimization

A 3-layer back-propagation neural network (BPNN) similar to that used in Reference^13^ was employed in this work for the transfer learning model. The network was composed of an input layer of 100 neurons corresponding to the 100 common selected features of each input spectrum; a hidden layer 5 neurons and an output layer with a single neuron corresponding to the targeted compound concentration. The function of the network was therefore to map an input spectrum (a vector of 100 dimensions) to a scalar which can be considered as the module of a vector in a hyperspace of 100 dimensions. The accuracy of the mapping was improved during the training process through different iteration loops under the supervision of the targeted concentration and using the model performance indication parameters specified above.

As shown in Fig. 5b, 3 hierarchized iteration loops, $i,j,k$, among them $i,j$ are doubled loops for a given $k$ ($\pm k$) surrounding the BPNN optimization loop performing the supervised optimization of the model.

- A doubled inner loop for $i = 1$ to 5: for the double cases of a given sample $k$ among the pellet samples being excluded ($- k$) or included ($+ k$) in the training data set, and a given data configuration $j$ of the rock spectra, the network was optimized within a cross-validation process where the model was trained using 4 ensemble of groups of replicate spectra, of for example, $i = 2,\;3,\;4,\;5$ with respectively 4, 4, 4 and 5 spectra for each sample. The resulted $REC(ij - k)$ and $REC(ij + k)$ were calculated for the respectively optimize d models for test $(ij - k)$ and $(ij + k)$. These models were then tested using the rest ensemble of groups of replicate spectra, $i = 1$ for instance, generating $RET(j - k)$ and $RET(j + k)$, together with the optimized models for test $(j - k)$ and $(j + k)$.
- A doubled intermediate loop for $j = 1$ to 3: in this loop, the above discussed loop $i$ was executed with 3 independent training rock data configurations for the 2 cases of a given sample *k* among the pellets being excluded from or included in the training data set. The model was further optimized. Corresponding calculation of *RET* resulted in $RET( - k)$ and $RET( + k)$.
- An outer loop for $k = 1$ to 18: in this loop the above discussed loop *i* and loop *j* were executed for each of the 18 training pellet samples successively assigned as the pellet *k*. For a given pellet *k*, $RET( - k)$ and $RET( + k)$ were compared. If an improvement was observed with the sample, it was kept in the final training sample set, otherwise it was rejected. This loop generated a model for test $(k)$ for each considered pellet sample with the corresponding $RET(k)$. The optimization process finally generated a model for validation with a minimized *RET* and *RMSET*.

#### Model validation

The resulted transfer learning model was validated by the pretreated spectra from the 12 validation rock samples including the 2 isolated rocks without counterpart pellet in the training sample set, with the identified features according to the common selected features between the training pellets and the training rocks. The parameters assessing the performance of the model for prediction, *REP*, *RMSEP* and *RSD* were calculated. These parameters indicate the performance of the model when used for predictions with LIBS spectra from rock samples, including unseen rocks, simulating thus a real application scenario. Remark that some of the training pellets, counterparts of the validation rocks and initially included in the model training sample set, were later rejected by the model training process (see Table 5 in the section “Methods”) and did not participate to the final model optimization process. Such configuration of validation provided the assessments of the transfer learning model in the both cases of rocks with counterpart pellets more or less seen during the model training and rocks totally unknown by the model.

## Results and discussion

### Analytical performances with the machine learning model

**Figure 6:**
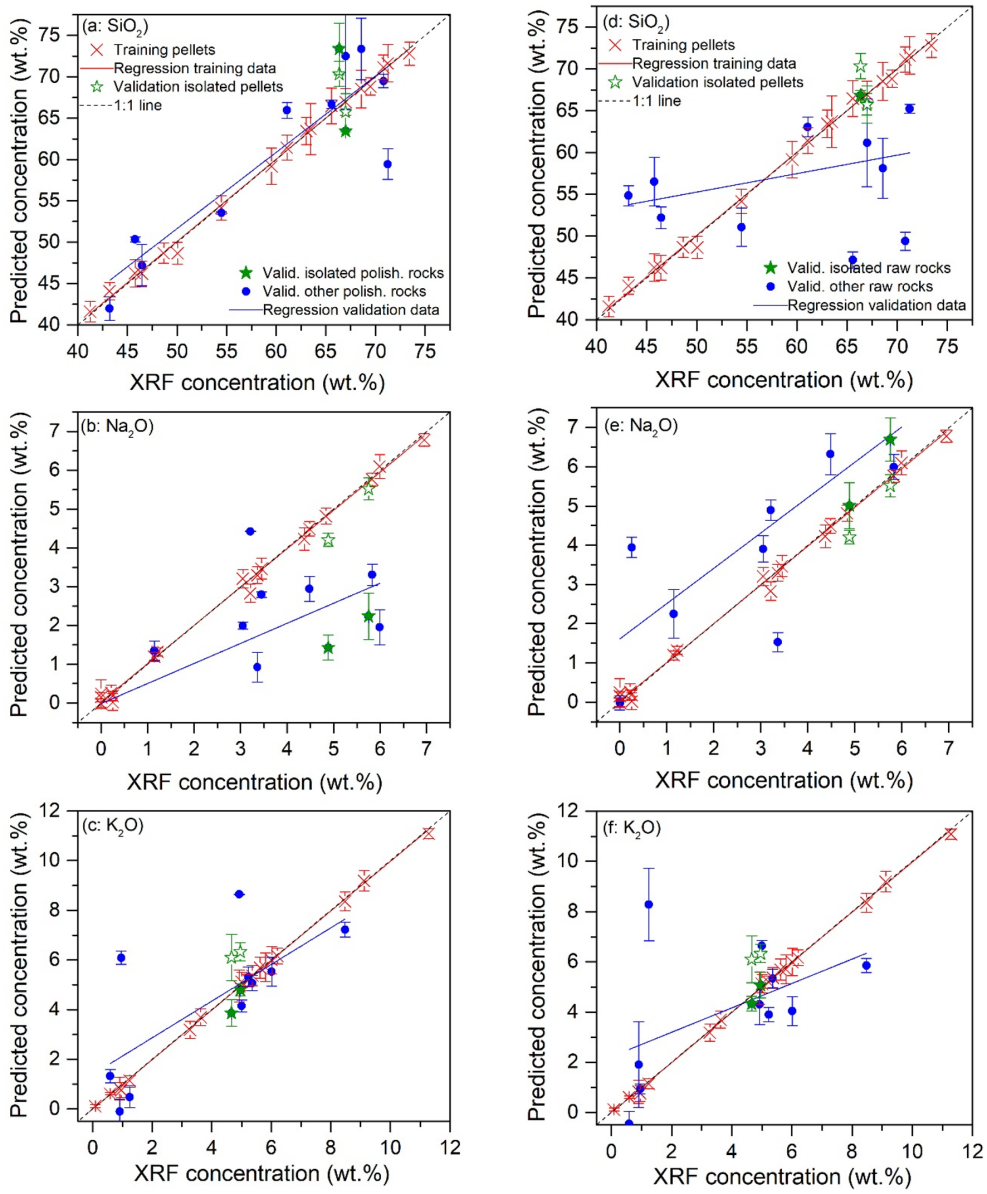
Presentation of the machine learning-based calibration models respectively for the 3 compounds SiO_2_ (**a**,**d**), Na_2_O (**b**,**e**) and K_2_O (**c**,**f**), with training data (red crosses) and their linear regressions (red lines); validation data of the 2 isolated pellets (open green stars); validation data of the validation polished (**a**–**c**) and raw (**d**–**f**) rocks (solid blue points) including isolated polished and raw rocks (solid green stars), together with their linear regressions (blue lines). Ground-truth diagonals are plotted in dotted lines in the figures as a reference for the models. The error bars of the presented data correspond to the standard deviations ($\pm SD$) of the calculated or predicted concentrations for a given training or validation sample. See the text for the details of standard deviation calculations.

In order to emphasize the improvement with transfer learning, we first present the results obtained with the machine learning models trained using the 18 training pellet samples and validated using the 2 isolated pellets and the 12 validation rocks respectively for the 3 concerned oxides, SiO_2_, Na_2_O and K_2_O. Such double validations allowed the correction of chemical matrix effect being explicitly checked with independent pellets before the check of physical matrix effect with rock samples. The training method described in Reference^13^ was implemented in this work to train a neural network. The training procedure was similar to the inner (loop *i*) and the intermediate (loop *j*) iteration loops used in the transfer learning model training (Fig. 5b) with a similar neural network structure. As shown in Fig. 3, the input variables were the 100 selected features in a pretreated spectrum of a training pellet sample for the training, and the 100 identified features in a pretreated spectrum of an isolated pellet sample or a validation rock sample for the validation. For the cross-validation optimization in the training process, similarly as in the transfer learning model training, $3 \times 5$-fold iterations were performed with 3 randomly organized pellet spectrum data configurations and 5 replicate groups of respectively 4, 4, 4, 4 and 5 spectra for each sample in a given data configuration. A larger number of data configurations did not lead to improvement of the model as shown by tests in our experiment. The training process was executed for the 3 concerned compounds resulting in 3 prediction models respectively for SiO_2_, Na_2_O and K_2_O. The obtained results for calibration and validation are shown in Fig. 6. In the figure, the training data are presented with their mean values obtained by averaging over the 15 individual values generated during the above mentioned $3 \times 5$-fold iterations, and the corresponding error bars representing the standard deviation ($\pm SD$) of the 15 individually calculated values. Similarly, the validation data are also shown with their mean values together with the error bars corresponding to $\pm SD$. The difference is however, that the individuals concerned by the statistics correspond to the ensemble of 21 pretreated spectra of each validation sample. Linear regressions are further performed respectively for the training and validation data (only for the 12 validation rocks), in order to measure the difference between them. Ground-truth diagonals are plotted in the figures as a reference for the models. The extracted parameters for assessment of model performances are presented in Table 2 according to the definitions provided above. Although in Fig. 6, the results are presented with a given typical pair of isolated validation samples (S2 and S6), in Table 2 the validation performances are calculated as average values of those obtained with 6 different pairs of isolated validation samples (S2 and S5; S1 and S6; S4 and S12; S15 and S16; S10 and S17; S9 and S20), which ensures the independence of these performances on the choice of validation samples. For validation with rocks, we make the distinction between the 2 isolated rocks and the 10 rocks with counterpart pellets.

**Table 2:** Parameters assessing the calibration and prediction performances of the machine learning models for SiO_2_, Na_2_O and K_2_O.

Assessment conditions		Assessment parameters	Compound			Average
			SiO_2_	Na_2_O	K_2_O	
Calibration	Pellets	$r^{2}$	0.997	0.996	0.999	0.997
		$Slope$	0.994	0.988	0.997	0.993
		$LOD\;(\% )$	5.14	0.62	1.01	2.26
		$REC\;(\% )$	5.61	9.84	3.75	6.40
		$RET\;(\% )$	7.42	10.5	5.20	7.71
		$RMSEC\;(wt.\% )$	1.61	0.84	0.75	1.07
		$RMSET\;(wt.\% )$	1.92	0.51	0.44	0.96
Validation	Pellets	$REP\;(\% )$	3.95	8.99	29.3	14.1
		$RMSEP\;(wt.\% )$	1.81	0.32	0.99	1.04
		$RSD\;(\% )$	2.76	4.59	10.5	5.95
	Polished rocks	$REP\;(\% )$	6.27	98.9	81.5	69.4
		$RMSEP\;(wt.\% )$	3.95	1.92	1.27	2.38
		$RSD\;(\% )$	182	28.5	56.9	89.1
	Polished rocks (isolated)	$REP\;(\% )$	13.2	55.4	57.8	42.1
		$RMSEP\;(wt.\% )$	8.13	1.16	1.83	3.71
		$RSD\;(\% )$	29.8	185	210	141
	Raw rocks	$REP\;(\% )$	13.8	176	82.3	90.7
		$RMSEP\;(wt.\% )$	8.11	1.57	1.48	3.72
		$RSD\;(\% )$	21.9	9.44	272	101
	Raw rocks (isolated)	$REP\;(\% )$	16.5	237	99.1	118
		$RMSEP\;(wt.\% )$	10.1	1.72	2.07	4.62
		$RSD\;(\% )$	23.2	133	164	107

In Fig. 6 and Table 2, we can see that the machine learning models trained with the training pellet samples present good calibration performances in terms of the usual assessment parameters including $r^{2}$, $LOD$, $REC$, $RET$, and $RMSE$. In addition, the validation with the 2 isolated pellet samples also show satisfactory results. This indicates an effective correction of the chemical matrix effect with machine learning regression, as we also observed in our previous works^12,13^. Whereas, we can remark an obvious degradation of the performance when the models were tested using the validation rock samples, in terms of $REP$, $RMSEP$ and $RSD$ due to the influence of the physical matrix effect. In Fig. 6, the 2 isolated rocks do not show a particularly “bad” behavior with respective to the other validation rocks with counterpart pellets in the training sample set, which would indicate the fact that the absence of bulk chemistry of a rock for the model training does not particularly further influence its prediction by the model. This remark is confirmed by Table 2. Moreover, Fig. 6 shows that the use of a model trained with pellet samples for prediction with the spectra from rock samples can lead to bias, with a shift of the linear regression of the validation data with respect to that of the training data, as well as variance, with a rotation of the linear regression of the validation data with respect to that of the training data. We can also remark that the model performance degradation observed with polished rock samples is in general, further aggravated for raw rock samples, as also indicated by Table 2 where we can see increased average $REP$ and $RMSEP$ when one passes from polished rocks to raw rocks. This means that the physical matrix effect arises due to different surface hardness and heterogeneity of a polished rock with respect to its corresponding pressed powder pellet. Surface roughness of a raw rock introduces additional perturbations leading to in general, larger prediction uncertainties. A detailed look on the validation performances in Table 2 however shows that the influence due to surface roughness (raw rocks) remains smaller than that due to surface hardness and heterogeneity (polished rocks), which contributes to the largest part of the physical matrix effect.

**Figure 7:**
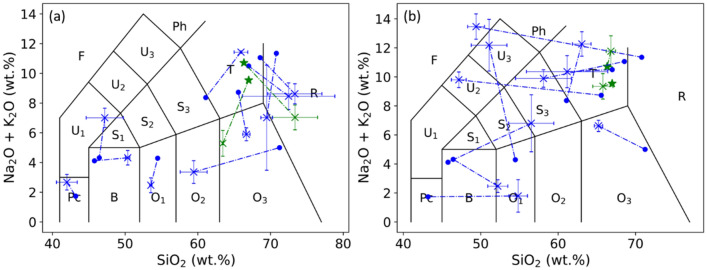
TAS classifications of the validation polished rock samples (**a**) and raw rock samples (**b**) using the machine learning models. The reference positions determined by XRF are presented in colored solid stars and circles respectively for the isolated rocks and the rest of the validation rocks, the corresponding model-predicted mean positions are presented with crosses in the same colors with associated error bars. A dashed line links the XRF reference position and the model-predicted position of a same rock sample. The error bars are calculated over the different pretreated spectra of a given sample.

As a consequence of the influence of the physical matrix effect, the TAS classification of the validation rock samples with the pellet machine learning models led to an unsatisfactory result as shown in Fig. 7. In this figure, the reference positions in the TAS diagram of the validation rock sample determined by their compositions measured using XRF (as shown in Fig. 1 and Table 4) are indicated with solid green stars for the 2 isolated rocks, and solid blue circular points for the rest of the validation rocks. The position predicted by the pellet machine learning models for the same sample is represented by a cross of the same color with error bars. More precisely, the cross represents the mean position calculated with the 21 pretreated replicate spectra of a sample. The error bars represent the standard deviations ($\pm SD$) of the concentrations over the replicate spectra, in particular the vertical error bars were obtained by summing the $SD$ s for the 2 concerned compounds. A dash-dot line further links the reference and the predicted positions of a same rock sample in order to explicitly indicate their correspondence. Such presentation thus allows calculating the rate of correct classification. If the pellet model-predicted mean position of a rock sample stays in the same TAS field as its XRF reference position, it is correctly classified. For the polished rock samples in Fig. 7a, we can see 3 correct classifications (S12, S17 and S20), corresponding to a correct classification rate of 25%. For the raw rock samples in Fig. 7b, we can see 4 correct classifications (S1, S2, S5 and S10), corresponding to a correct classification rate of 33.3%. Here we can see an even lower rate of correct classification for polished rocks, confirming a dominant contribution to the physical matrix effect by a change of sample surface hardness and heterogeneity, comparing to the influence of surface roughness. An additional remark concerns the 2 isolated rocks, even though both of them were correctly classified in the case of raw rocks, we still cannot conclude on their particularity with respect to the rest of validation rocks due to their absence of counterpart pellet in the training sample set.

### Analytical performances with the transfer learning model

**Figure 8:**
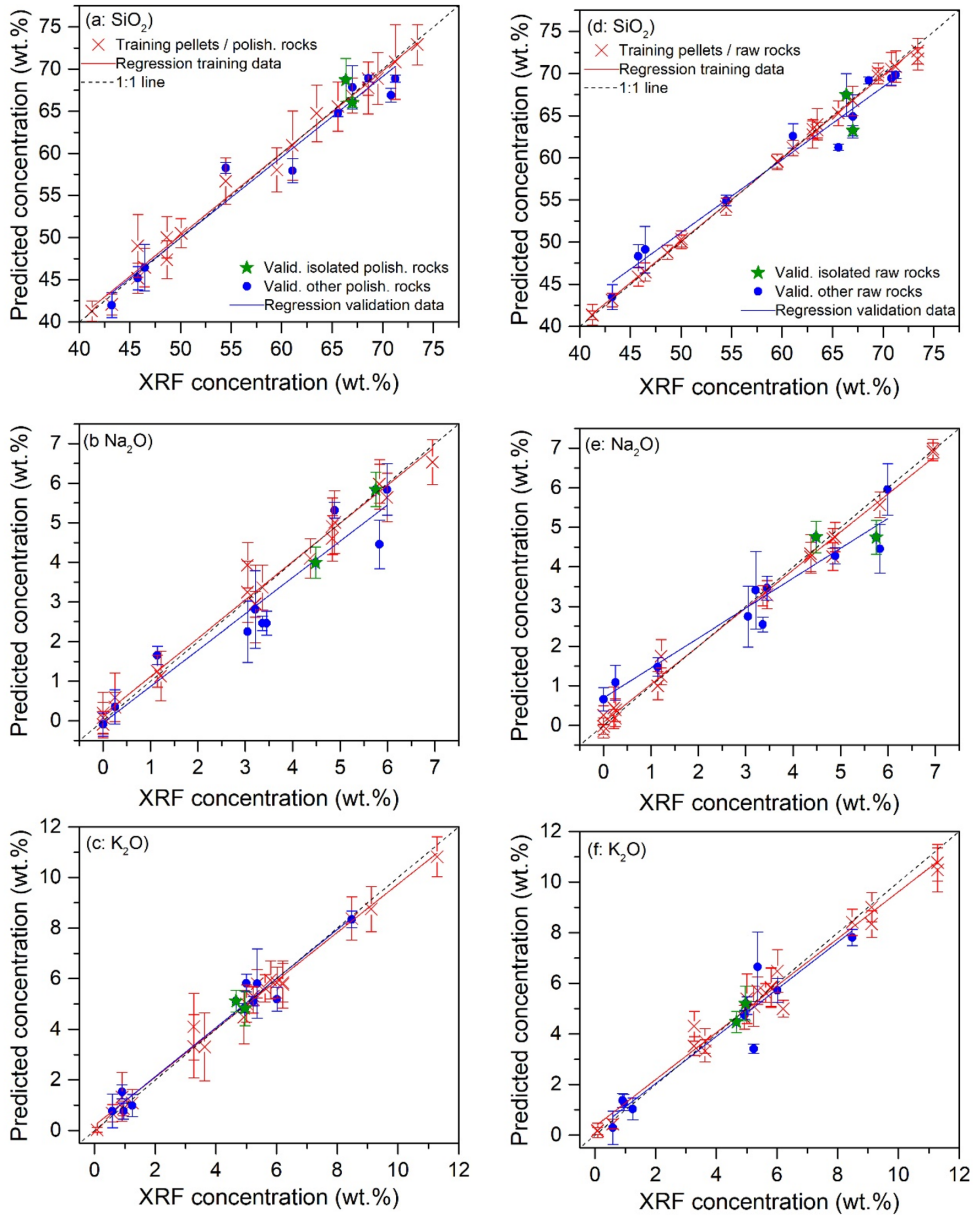
Presentation of the transfer learning-based calibration models respectively for the 3 compounds SiO_2_ (**a**,**d**), Na_2_O (**b**,**e**) and K_2_O (**c**,**f**), with training data (red crosses) and their linear regressions (red lines); validation data for the validation polished (**a**–**c**) and raw (**d**–**f**) rocks (solid blue points) including isolated polished and raw rocks (solid green stars), together with their linear regressions (blue lines). Ground-truth diagonals are plotted in dotted lines in the figures as a reference for the models. The error bars of the presented data correspond to the standard deviations ($\pm SD$) of the calculated or predicted concentrations for a given training or validation sample. See the text for the details of standard deviation calculations.

Calibration models resulting from transfer learning are shown in Fig. 8 with a similar format as those resulting from machine learning presented in Fig. 6, in order to review the improvements by comparison. The extracted parameters for assessment of the model performances are presented in Table 3 with validation performances calculated as average values of those obtained with the 6 different pairs of isolated validation samples. In Fig. 8, we can see significant reductions of bias and variance of the predicted concentrations for the validation rock samples including the 2 isolated rocks, with respect to the training data of a part of the pellet samples and the training rock samples. In particular, for SiO_2_, 14 pellet samples were retained in the final training sample set among the 18 ones by the optimization loop during the model training process to combine with the training rock samples in the both cases of polished and raw rocks. For Na_2_O or K_2_O, the retained pellet samples are respectively 15 or 15 and 13 or 14 in the both cases of polished and raw rock samples. The details of the pellet samples retained in the training sample sets are shown in Table 5 in section “Methods”. In this table, we can remark that the counterpart pellets of the 8 training rock samples are often retained in the final training sample set. This means that samples with a same chemical composition but different physical forms are more appreciated for an efficient training of a transfer learning model. At the same time, some counterpart pellets of validation rock samples are rejected by the optimization process. In Fig. 8, we do not remark particular behavior for the 2 isolated rocks with respect to the other validation rocks as in Fig. 6. In Table 3, we can see that although the transfer learning models present in general, slightly lower calibration performances in terms of $r^{2}$, $LOD$, $REC$, $RET$ and $RMSE$ compared to the machine learning models, the prediction performance for polished and raw rock samples are much improved, especially for $REP$ and $RMSEP$. This means that the participation of the 8 rock samples in the training data set together with the retained pellet samples with common selected features, effectively takes into account the physical matrix effect and reinforces the robustness of the models for prediction for rock samples, including isolated rocks totally unknown by the models. We remark in particular, the prediction performances for both polished and raw rocks are simultaneously improved, showing the effectiveness of the transfer learning models in the correction of physical matrix effects of different origins.

**Table 3:** Parameters assessing the calibration and prediction performances of the transfer learning calibration models for SiO_2_, Na_2_O and K_2_O.

Assessment conditions		Assessment parameters	Compound			Average
			SiO_2_	Na_2_O	K_2_O	
**Pellets/polished rocks**	Calibration	$r^{2}$	0.994	0.985	0.994	0.993
		$Slope$	0.989	1.017	0.993	0.999
		$LOD\;(\% )$	4.53	2.03	2.45	3.00
		$REC\;(\% )$	1.52	15.1	10.4	9.01
		$RET\;(\% )$	1.90	15.71	9.72	9.11
		$RMSEC\;(wt.\% )$	2.61	0.75	0.80	1.39
		$RMSET\;(wt.\% )$	2.40	0.62	0.82	1.28
	Validation	$REP\;(\% )$	2.76	20.6	16.4	13.3
		$RMSEP\;(wt.\% )$	1.68	0.53	0.35	0.85
		$RSD\;(\% )$	128	45.8	47.5	73.8
	Validation (isolated)	$REP\;(\% )$	6.07	27.4	11.3	14.9
		$RMSEP\;(wt.\% )$	3.48	0.89	0.17	1.51
		$RSD\;(\% )$	11.76	37.0	49.4	32.7
**Pellets/raw rocks**	Calibration	$r^{2}$	0.998	0.993	0.997	0.996
		$Slope$	0.985	0.961	0.993	0.980
		$LOD\;(\% )$	3.70	1.03	2.26	2.33
		$REC\;(\% )$	5.61	15.7	6.40	9.24
		$RET\;(\% )$	4.90	16.2	7.72	9.60
		$RMSEC\;(wt.\% )$	2.44	0.75	0.88	1.36
		$RMSET\;(wt.\% )$	2.37	0.49	0.72	1.19
	Validation	$REP\;(\% )$	3.11	42.0	19.8	21.6
		$RMSEP\;(wt.\% )$	1.85	0.54	0.50	0.96
		$RSD\;(\% )$	24.1	21.3	101	48.8
	Validation (isolated)	$REP\;(\% )$	1.68	21.5	10.2	11.1
		$RMSEP\;(wt.\% )$	1.00	0.66	0.19	0.62
		$RSD\;(\% )$	13.2	25.4	67.3	35.3

**Figure 9:**
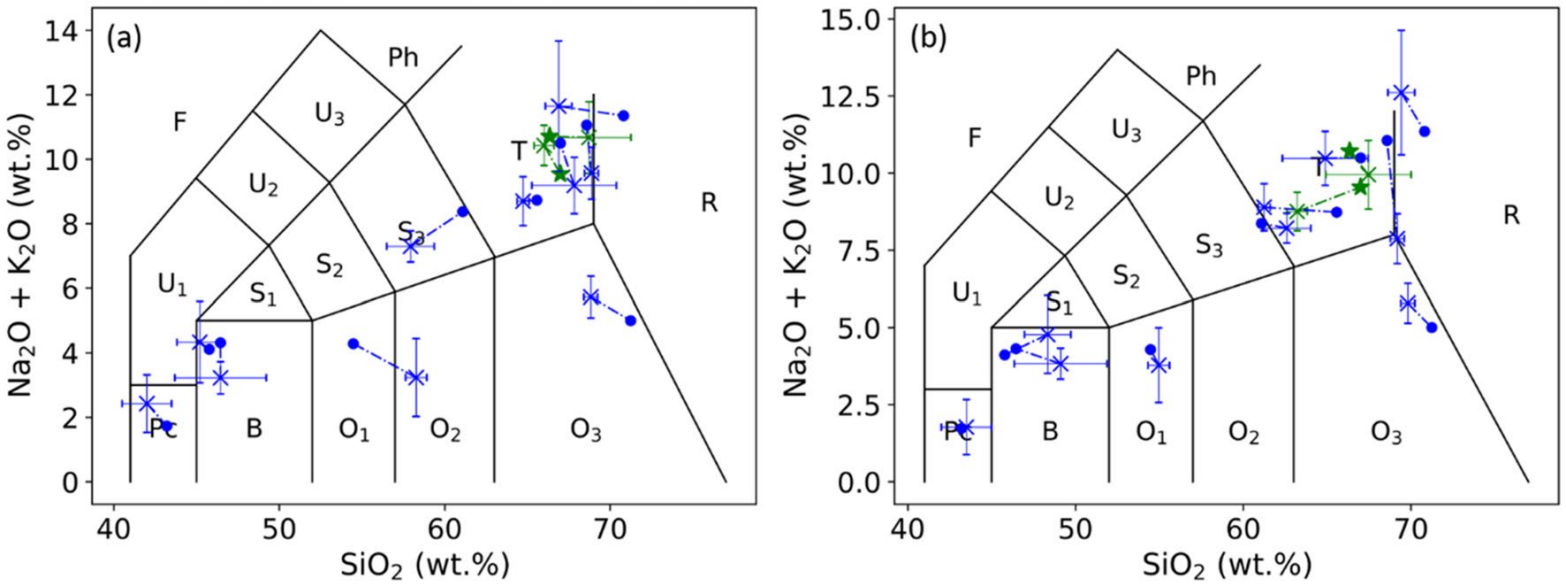
TAS classification using transfer learning models of the validation rock samples, (**a**) for polished rocks and (**b**) for raw rocks. The same symbols are used as in Fig. 7.

The calibration models shown in Fig. 8 were used to present the validation rock samples in a TAS diagram. The obtained results are shown in Fig. 9a for polished rock samples and Fig. 9b for raw rock samples using the same symbols as in Fig. 7. We can see a much improved result conforming the good performances of the transfer learning models shown in Fig. 8 and Table 3. A detailed counting shows 10 correctly classified validation samples for the both polished and raw rocks, including the 2 isolated rocks. Only two samples were classified into a wrong field (S12 and S15 for polished rocks, S4 and S6 for raw rocks). The rate of correct classification can thus be determined to be 83.3% in the both cases. These results show the effectiveness of the developed method to correct the physical matrix effect. Confirming the observation in Fig. 8, no particular behavior can be observed for the 2 isolated rocks in the both cases of polished and raw rocks with respect to the other validation rocks.

## Conclusions

In this work, within a given application of classification of rocks using the TAS diagram, we have introduced transfer learning in LIBS spectral data treatment to improve the performance of the models trained using laboratory standard samples in the form of pressed powder pellet, when used for prediction with LIBS spectra acquired from natural rocks with a polished surface or in a raw state. Such scenario corresponds to the important application of analysis of rocks with LIBS on Mars, although the used experimental configuration compared to the current rovers on Mars remains still quite different, concerning the ambient gas, the laser excitation, as well as the spectrum detection. The purpose was therefore to work on a general method that can be later implemented according to specific experimental conditions into particular applications. More precisely, feature-representation-transfer and instance-transfer as the two important processes of transfer learning were implemented in the LIBS spectral data treatment. The performances of the resulted transfer learning models were compared with those of the machine learning models. Significant improvements have been realized for prediction with LIBS spectra acquired on polished and raw rock samples for the 3 concerned compounds involved in the TAS classification, SiO_2_, Na_2_O and K_2_O. The rate of correct TAS classification has been improved from 25% for polished rocks and 33.3% for raw rocks with the machine learning models to 83.3% for the both types of rock samples with the transfer learning models. The obtained results therefore demonstrate the effectiveness of transfer learning to overcome the physical matrix effect due to the change of sample physical state in LIBS analyses.

There are still steps forward to realize in research and development to apply the method developed in this work to Mars explorations with LIBS. Such steps should involve a larger set of samples, with the possibility to isolate more rock samples for the independent validation of the transfer learning models, although the results shown in this work do not reveal obviously different behavior of the isolated validation rocks with respect to the other validation rocks that can have a counterpart pellet in the model training sample set. This would indicate a dominant physical matrix effect in the given configuration of study. It is also to be taken into account the experimental conditions, including the measurement environment (ambient gas and its pressure), the used laser parameters and the spectrum detection, in order to reduce the dissimilarities between a laboratory simulation experiment and the in situ LIBS measurements on Mars to a strict minimal related to the lack of complete knowledge about a real sample to be analyzed on Mars. Beyond analysis of rocks with LIBS in Mars explorations, our findings in this work can also have more general interests in the development of LIBS technique for various applications involving sets of samples with different surface physical properties.

## Methods

### Detailed chemical compositions of the samples

The detailed chemical compositions of the rocks studied in this work are shown in Table 4.

**Table 4:** Compositions in wt.% of the samples used in the experiment determined using XRF, together with their corresponding TAS fields.

Geological name	Sample mane	Oxide composition (wt. %)								Field in TAS (cf. Fig. 1)
		SiO_2_	Fe_2_O_3_	Al_2_O_3_	CaO	MgO	K_2_O	Na_2_O	Others	
Granite-porphyry	S1	66.99	2.39	16.04	1.98	0.74	6.01	4.48	1.37	T
Monzonitic-granite	S2	66.34	1.94	17.11	2.19	0.50	4.96	5.75	1.21	T
Plagiogranite	S3	63.47	1.98	18.79	3.17	0.52	3.63	6.95	1.49	T
Moyite	S4	68.57	1.45	16.50	1.42	0.24	5.23	5.83	0.76	T
Biotite admellite	S5	66.99	2.09	16.18	2.93	1.17	4.66	4.88	1.10	T
Shiying syenite	S6	61.08	6.85	14.23	5.05	1.66	4.92	3.45	2.76	S_3_
Granogabbro	S7	48.65	13.72	14.84	5.89	3.84	3.27	4.84	4.95	U_2_
Basaltglass	S8	50.05	15.00	14.82	6.24	3.73	6.21	1.22	2.73	S_2_
Mudstone	S9	65.57	3.81	18.33	0.31	2.18	8.48	0.25	1.07	T
Shale	S10	71.24	3.29	15.94	1.07	1.44	5.00	0.00	2.02	O_3_
Aleuritic texture shale	S11	59.53	13.62	17.32	0.49	1.57	5.62	0.00	1.85	O_2_
Packsand	S12	54.47	4.88	11.41	18.52	4.43	1.24	3.05	2.00	O_1_
Siltstone	S13	69.48	1.31	15.05	0.37	0.50	11.29	0.22	1.78	R
Marl	S14	63.04	5.57	17.73	0.91	2.05	9.12	0.25	1.33	T
Conglomerate	S15	70.80	1.41	14.87	0.79	0.16	5.36	5.99	0.62	R
Gabbro	S16	46.45	11.04	18.15	10.73	7.96	0.96	3.36	1.35	B
Angle flash green mudstone	S17	45.77	17.77	13.58	8.66	6.08	0.91	3.21	4.02	B
Module rock	S18	71.01	1.57	16.88	0.29	0.38	5.98	3.22	0.67	R
Red granite	S19	73.42	1.09	13.55	0.64	0.62	5.80	4.37	0.51	R
Pyroxenite	S20	43.20	15.92	8.48	14.09	14.09	0.59	1.14	2.49	Pc

### Feature selection

**Figure 10:**
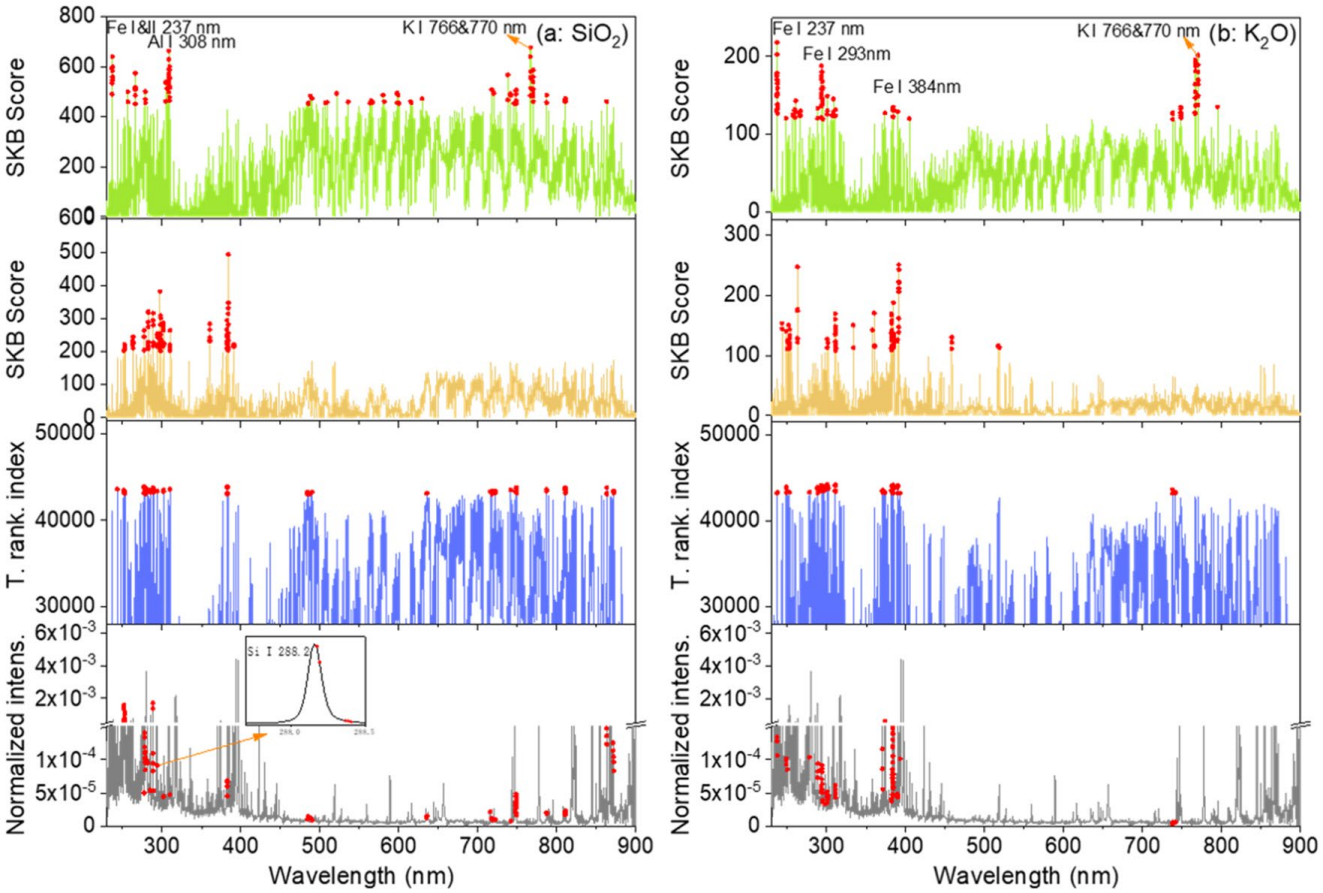
Results of feature selection for the pair of sample types pellets/raw rocks: for (**a**) SiO_2_ and (**b**) K_2_O. Detailed captions can be found with Fig. 4.

**Figure 11:**
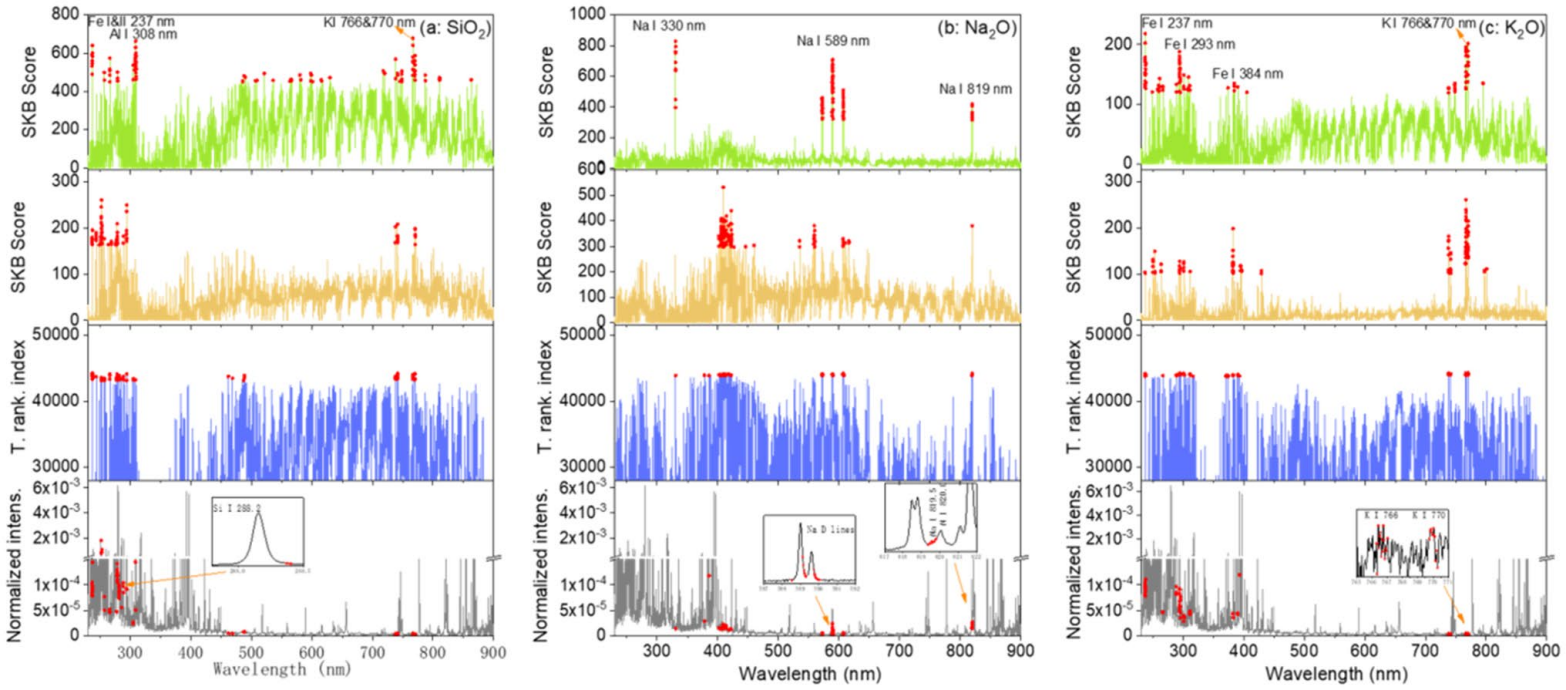
Results of feature selection for the pair of sample types pellets/polished rocks: for (**a**) SiO_2_, (**b**) Na_2_O and (**c**) K_2_O. Detailed caption can be found with Fig. 4.

The results of feature selection for K_2_O and SiO_2_ for the pair of sample types pellet/raw rocks, are shown in Fig. 10. The results of feature selection for Na_2_O, K_2_O and SiO_2_ for the pair of sample types pellet/polished rocks, are shown in Fig. 11.

### Instance-transfer

The information about the pellet samples retained in the final training sample set after the optimization loop of transfer learning model is presented in Table 5.

**Table 5:** Pellet samples retained in the final transfer learning training sample set by the optimization loop in the model training process. The counterpart pellets of the 8 training rock samples are highlighted in bold.

Sample form	Compound	Nb. of sample retained over the 18 initial ones	Names of the retained pellet samples
Polished rocks	SiO_2_	14	**S3**, S6, **S7**, **S8**, S9, S10, S**11**, **S13**, **S14**, S15, S17, **S18**, **S19**, S20
	Na_2_O	15	**S3**, S4, S6, **S7**, **S8**, S9, S10, **S11**, S12, **S13**, **S14**, S16, **S18**, **S19**, S20
	K_2_O	15	S1, **S3**, S4, S6, **S7**, **S8**, S9, **S11**, S12, **S13**, **S14**, S15, **S18**, **S19**, S20
Raw rocks	SiO_2_	14	**S3**, S6, **S7**, **S8**, S9, S10, **S11**, S12, **S13**, **S14**, S15, S17, **S19**, S20
	Na_2_O	13	S6, **S7**, S9, S10, **S11**, S12, **S13**, **S14**, S16, S17, **S18**, **S19**, S20
	K_2_O	14	S1, **S3**, S4, S6, **S7**, **S8**, S9, **S11**, S12, **S13**, S16, **S18**, **S19**, S20

## Data Availability

The LIBS spectral data treated in this work are available under request by readers for suitable reasons.

## Code availability

The program was written using Python version 3.7 and the BPNN was implemented using Keras framework based on TensorFlow 1.14.0. Scikit-learn and NumPy were used. In addition, Origin Pro 8.0 (Origin Lab Corporation, Northampton, MA, USA) was used to draw the figures. All the programs were run on a PC (CPU: Intel(R) Xeon(R) Silver 4214 @2.20 GHz, RAM: 128.00 GB) under Windows 10.
